# Closely Spaced MEG Source Localization and Functional Connectivity Analysis Using a New Prewhitening Invariance of Noise Space Algorithm

**DOI:** 10.1155/2016/4890497

**Published:** 2015-12-24

**Authors:** Junpeng Zhang, Yuan Cui, Lihua Deng, Ling He, Junran Zhang, Jing Zhang, Qun Zhou, Qi Liu, Zhiguo Zhang

**Affiliations:** ^1^Department of Medical Information Engineering, School of Electrical Engineering and Information, Sichuan University, Chengdu 610065, China; ^2^School of Chemical and Biomedical Engineering, School of Electrical and Electronic Engineering, Nanyang Technological University, Singapore 639798; ^3^School of Humanities and Information Management, Chengdu Medical College, Chengdu 610083, China

## Abstract

This paper proposed a prewhitening invariance of noise space (PW-INN) as a new magnetoencephalography (MEG) source analysis method, which is particularly suitable for localizing closely spaced and highly correlated cortical sources under real MEG noise. Conventional source localization methods, such as sLORETA and beamformer, cannot distinguish closely spaced cortical sources, especially under strong intersource correlation. Our previous work proposed an invariance of noise space (INN) method to resolve closely spaced sources, but its performance is seriously degraded under correlated noise between MEG sensors. The proposed PW-INN method largely mitigates the adverse influence of correlated MEG noise by projecting MEG data to a new space defined by the orthogonal complement of dominant eigenvectors of correlated MEG noise. Simulation results showed that PW-INN is superior to INN, sLORETA, and beamformer in terms of localization accuracy for closely spaced and highly correlated sources. Lastly, source connectivity between closely spaced sources can be satisfactorily constructed from source time courses estimated by PW-INN but not from results of other conventional methods. Therefore, the proposed PW-INN method is a promising MEG source analysis to provide a high spatial-temporal characterization of cortical activity and connectivity, which is crucial for basic and clinical research of neural plasticity.

## 1. Introduction

Magnetoencephalography (MEG) is becoming a more and more popular brain imaging tool for exploring brain dynamics and interactions because of its millisecond temporal precision and high spatial resolution [1]. Particularly, the high temporal-spatial resolution of MEG enables tracking of dynamic neuronal interactions, which is crucial to study neural plasticity [2–5]. For example, surgical resection is a necessary operation for serious brain tumors-II gliomas, and it is important to precisely localize resection regions to optimize the benefit/risk ratio of the surgery. The variability of normal anatomy and the functional reorganization due to cerebral plasticity phenomena make classic anatomic boundaries insufficient for predicting associated function. The emerging technology of individual brain mapping and functional connectivity (FC) can individually generate a functional map and facilitate localization of functional boundaries, which will greatly increase the accuracy in surgical resection (for a review, see [4]). Also, Tarapore et al. suggested that MEG-based FC was a better predictor of long-term postoperative morbidity than intraoperative electrical stimulation [6]. In such a case, neural plasticity plays an important role in postoperative brain tissue and function development and FC can provide reliable indicators of neural plasticity related to postoperative neural development. In addition, the study of MEG-based FC is useful for understanding the short-term plasticity associated with sleep and memory. For example, generators of the oscillatory regime and FC underlying early and late synchrony may help understand the role of sleep spindles in brain plasticity [7].

Generally, MEG-based brain connectivity analysis can be conducted at two levels: sensor level and source level. Interpretation of sensor-level connectivity is not straightforward, as it suffers from a low spatial resolution and is severely corrupted by effects of field spread [8]. To overcome the limitation of sensor-level connectivity analysis, it is more desired to estimate connectivity among cortical sources. Source-level connectivity analysis generally comprises two steps: source localization and connectivity analysis [9]. Firstly, the locations of MEG sources and their time courses will be estimated using a spatiotemporal source model from scalp waveforms to mitigate the intrinsic volume conduction (field spread) effect. Second, connectivity measures (such as correlation and phase synchronization) will be estimated from time courses of cortical sources. Therefore, correct estimates of source locations and their time courses are the prerequisites of source-level connectivity analysis.

Many source localization methods, such as the linear constraint minimum variance (LCMV) beamformer [10, 11], and sLORETA (standardized low resolution brain electromagnetic tomography) [12] have been widely used to identify source locations from MEG and to reconstruct time courses in source space. However, these classical source localization methods have difficulty in resolving sources with strong connectivity (i.e., source time courses are highly correlated), especially when underlying sources are closely spaced. As shown in [8], previous work have already attempted to resolve correlated sources, such as [13, 14]. However, the distance between sources is generally randomly set in simulations of these papers. A close distance between two sources will add the difficulty in sources localization, because two sources have very similar lead field. Apparently, inaccurate results of source localization will lead to distorted reconstruction of source time courses and then will adversely affect sequential source connectivity analysis. In our previous paper, a new source localization method, invariance of noise space (INN) [15], has been proposed for MEG source localization. Based on the fact that modulations of source strengths only change the variance in signal subspace but do not change that in noise subspace, the INN method can provide more accurate results than conventional source localization methods, such as LCMV beamformer and MUSIC. In particular, INN has better performance in dealing with sources with strong interaction, even in the case that multiple sources are close to each other [15]. However, the simulation study in [15] only tested the performance of INN under simulated Gaussian noise and it is still not clear whether the INN method can work well under real-world MEG noise. Actually, MEG noise has different properties with Gaussian noise. Real MEG noise, as a kind of spatially correlated noise, is a combination of SQUID (superconducting quantum interference device) noise and interference generated by both environmental and biomagnetic sources of no interest. While a Gaussian model may be adequate for describing SQUID noise, the interference will necessarily be correlated across MEG sensors and will not exhibit a flat frequency spectrum as white noise does. Therefore, it is necessary to examine the effectiveness of the INN method in source localization and subsequent source connectivity analysis in a real noise environment. More importantly, correlated noise usually deteriorates performance of source localization, so it is desirable to further improve the INN method to make it robust under real-world correlated noise.

In this study, we first intensively investigate the performance of INN in identifying sources, under the condition of simulated white noise as well as real spontaneous MEG noise. We particularly focus on the cases where sources are closely spaced and highly correlated. Further, to alleviate the effect of the correlated noise on localization performance of INN, we proposed a new prewhitening INN (PW-INN) method, which can suppress correlated noise by projecting MEG data to a new space defined by the orthogonal complement of dominant eigenvectors of correlated MEG noise. Next, based on identified source locations using PW-INN, source time courses can be derived using the classical least squares method. Finally, we used phase synchronization (PS) to measure the FC between source time courses estimated using PW-INN. For comparison, the classical LCMV beamformer and sLORETA are also tested on simulated MEG data in terms of their performance in identification of source locations, reconstruction of source time courses, and inference of source connectivity.

## 2. Methods and Materials

### 2.1. Methods

#### 2.1.1. Problem Formulation

The MEG data **Y**(*t*) generated by current dipole sources can be modeled as

(1) $$\begin{array}{c} Y\left(\;t\;\right)=AX\left(\;t\;\right)+n\left(\;t\;\right), \end{array}$$

where **A** is the gain matrix relating the measured signals to the dipole amplitudes, rows of **X**(*t*) are the time courses of the current dipoles, and **n**(*t*) is additive noise.

Assuming that **n**(*t*) is uncorrelated across the channels, that the variance of the noise on each channel is *σ* ^2^, and that the signal and noise are uncorrelated, the correlation matrix of the MEG data is

(2) $$\begin{array}{c} R=\langle \;Y\left(\;t\;\right)Y{\left(\;t\;\right)}^{T}\;\rangle =APA^{T}+\sigma^{2}I, \end{array}$$

where **P** = 〈**X**(*t*)**X**(*t*)^*T*^〉. Based on this assumption of uncorrelated noise, many source localization methods have been developed. However, this assumption does not hold true for real MEG signals. In the following, we will first introduce classical source localization methods, LCMV beamformer and sLORETA, and then develop a new source localization method to deal with real-world correlated noise.

#### 2.1.2. Beamformer

Beamformers, as adaptive spatial filters, pass the signal from desirable locations while blocking signals from other locations. The source activity **s**(*θ*, *t*) at location *θ* and time *t* is estimated by a simple linear operation,

(3) $$\begin{array}{c} s\left(\;\theta ,t\;\right)=W{\left(\;\theta \;\right)}^{T}Y\left(\;t\;\right), \end{array}$$

where **W**(*θ*) is a column vector consisting of a set of spatial filter weights. In an LCMV beamformer, **W**(*θ*) minimizes the variance of the filter output:

(4) minw WθTRWθsubject  to WθTaθ=1,

where **a**(*θ*) indicates the gain matrix at location *θ*. The solution of this constrained optimization problem [10, 11] is

(5) $$\begin{array}{c} W\left(\;\theta \;\right)=R^{-1}a\left(\;\theta \;\right){\left[\;a{\left(\;\theta \;\right)}^{T}R^{-1}a\left(\;\theta \;\right)\;\right]}^{-1}. \end{array}$$

Mapping the filter output as a function of location generates functional (pseudo) image. In this study, we use a vector LCMV beamformer described in previous studies [10, 11].

#### 2.1.3. sLORETA

The sLORETA [12] is based on Minimum Norm Estimation (MNE) [16] and it standardizes the source distribution estimated from MNE by the variance of each estimated dipole source. The solution of MNE at location *θ* and time *t* can be written as

(6) $$\begin{array}{c} s{\left(\;\theta ,t\;\right)}_{MNE}=A^{T}{\left(\;A^{T}A+\beta I\;\right)}^{-1}Y\left(\;\theta ,t\;\right), \end{array}$$

where *β* > 0 is a scalar regularization parameter to be chosen allowing inversion of the matrix in parenthesis (Tikhonov regularization) and **I** is the identity matrix. In order to obtain sLORETA solution, **s**(*θ*, *t*)_MNE_ is normalized by its estimated variance **σ** assuming independence of source activity, defined as **σ** = **A** ^*T*^(**A** ^*T*^ **A** + *β* **I**)^−1^ **A**. Then, the sLORETA solution at source grid points *θ* at time *t* is

(7) $$\begin{array}{c} s{\left(\;\theta ,t\;\right)}_{sLORETA}=s{\left(\;\theta ,t\;\right)}_{MNE}^{T}\sigma_{\theta }^{-1}s{\left(\;\theta ,t\;\right)}_{MNE}. \end{array}$$

#### 2.1.4. INN

In [15], we developed a new INN method, which is based on the assumption that the noise subspace of a multidimensional signal is invariant with respect to the strengths of the sources. Let us define a matrix **D** ^*θ*^ as

(8) $$\begin{array}{c} D^{\theta }=R+ha\left(\;\theta \;\right)a{\left(\;\theta \;\right)}^{T}, \end{array}$$

where **R** is the data correlation matrix of (2), **a**(*θ*) is the lead field matrix generated by a unit source at location *θ*, and *h* is a positive constant scalar. The cost function of INN is

(9) $$\begin{array}{c} J\left(\;\theta \;\right)=\frac{1}{\sum_{i=p+1}^{K}\left(\mu_{i}^{\theta }-\lambda_{i}\right)}, \end{array}$$

where *λ*_*i*_ is ordered singular values of **R**, *μ*_*i*_^*θ*^ is the ordered singular values of **D**(*θ*), *p* indicates the number of sources, and *K* indicates the number of rows or columns of **D**(*θ*). As (9) implies, if one probe source is exactly placed at one of tentative source locations, only the variance of signal space of **D** ^*θ*^ will increase and the noise space keeps unchanged. As a result, the cost function *J*(*θ*) will generate a peak since the denominator in (8) is approximately equal to zero. On the other hand, if one probe source is placed at locations other than true source locations, the noise space of **D** ^*θ*^ will correspondingly change. Then, *J*(*θ*) will obtain a small value since the values of the denominator in (8) will deviate from zero. The values of the cost function *J*(*θ*) can be used as imaging indices to generate pseudoimages, and the peaks of *J*(*θ*) could be regarded as the locations of the sources.

#### 2.1.5. Prewhitening INN (PW-INN)

LCMV beamformer, sLORETA, and INN are all based on the assumption of uncorrelated noise, which is actually not true for real MEG signals. Generally, MEG noise is correlated between MEG channels. Denoting the additive correlated noise as **n**_*c*_(*t*), then (1) becomes

(10) $$\begin{array}{c} Y\left(\;t\;\right)=AX\left(\;t\;\right)+n_{c}\left(\;t\;\right). \end{array}$$

By singular value decomposition of the noise covariance matrix **R**_*c*_ = 〈**n**_*c*_(*t*)**n**_*c*_(*t*)^*T*^〉, we obtain *M* dominant left eigenvectors, **S**_*i*_, *i* = 1,…, *M*. Here, the first *M* eigenvectors, accounting for most of total noise variance (e.g., 90%), were chosen to construct a matrix [**S**_1_, **S**_2_,…, **S**_*M*_]. The orthogonal complement matrix of [**S**_1_, **S**_2_,…, **S**_*M*_], indicated by **P**, can be calculated according to

(11) $$\begin{array}{c} P=I-\left(\;S_{1}S_{1}^{T}+S_{2}S_{2}^{T}+\cdots +S_{M}S_{3}^{T}\;\right). \end{array}$$

Multiplying **P** to two sides of (1), we get

(12) $$\begin{array}{c} PY\left(\;t\;\right)=PAX\left(\;t\;\right)+Pn_{c}\left(\;t\;\right). \end{array}$$

It is straightforward that **n**_*c*_(*t*) can be decomposed into

(13) $$\begin{array}{c} n_{c}\left(\;t\;\right)\approx S_{1}V_{1}^{T}+S_{2}V_{2}^{T}+\cdots +S_{M}V_{M}^{T}, \end{array}$$

where **V**_*i*_ *i* = 1,…, *M*, indicates the components along the directions **S**_*i*_ *i* = 1,…, *M*, respectively. By multiplying (11) and (13), we can readily get **P** **n**_*c*_(*t*) ≈ 0. That is, by projecting correlated signals to the space defined by **P**, the second term of the right side of (12) almost disappears. Then, the conventional INN method can be well applied to the projected MEG data (i.e., **P** **Y**(*t*)) and projected lead field **P** **a**(*θ*). By this means, the new PW-INN method can largely suppress correlated noise to overcome the limitation of INN in presence of real-world correlated noise. Once source locations are identified using PW-INN, source time courses can easily be derived using the least squares method [17].

### 2.2. Simulations

#### 2.2.1. Model Configuration and Parameter Definition

**Figure 1:**
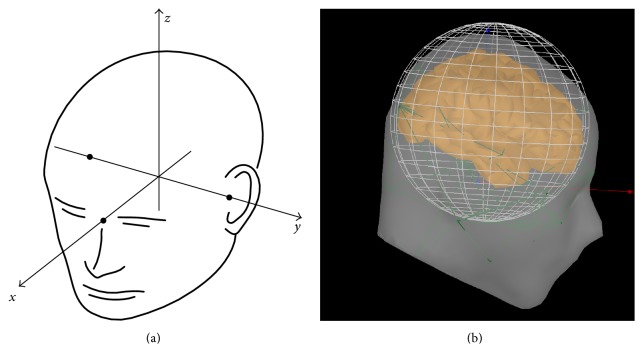
Simulation settings. (a) Coordinate system used in the simulation settings. The *x*-axis of the head coordinate system passes through the two periauricular points with positive direction to the right. The *y*-axis passes through the nasion and is normal to the axis. The *z*-axis points up according to the right-hand rule and is normal to the *xy* plane. (b) Illustration of the head model, where the whole brain was completely encompassed by the sphere.

In the simulations, the single layer sphere head model was adapted. The sensor array comprised 272 magnetometers arranged in an array on a sphere with 100 mm radius. The average distance between sensors was 22 mm. Our coordinate system is defined in Figure 1(a) and the whole brain was completely encompassed by the sphere, as shown in Figure 1(b), and thus the brain volume was well modeled. The brain volume was partitioned into about 17,000 grid points and the distance between neighboring grid points is 5 mm. Volume source space was used in this study and the lead field was calculated using NUTMEG [18, 19]. The signal-to-noise ratio (SNR) was defined as the ratio of the Frobenius norm of the data matrix to that of the noise matrix. Two types of noise were employed in simulations: white noise and real spontaneous MEG recordings. The spontaneous MEG data used as simulated noise were collected from a passive pure-tone listening task and extracted from a prestimulus period. MEG data were acquired with a 275-channel whole-head MEG device from CTF Systems. The sampling frequency was set at 1200 Hz. The selected prestimulus MEG data included samples at 120 time points from −100 ms to 0 ms (stimulus onset). Prestimulus MEG trials from one subject were used as real MEG noise and added to simulated MEG signal. In PW-INN, the prestimulus MEG data used to construct the projection matrix are randomly selected from trials of the same subject that are different from those trials used to simulate real MEG noise. Correlation coefficient (*r* ^2^) was used to measure the degree of linear correlation between two source time courses.

#### 2.2.2. Resolvability of Closely Spaced Sources

**Figure 2:**
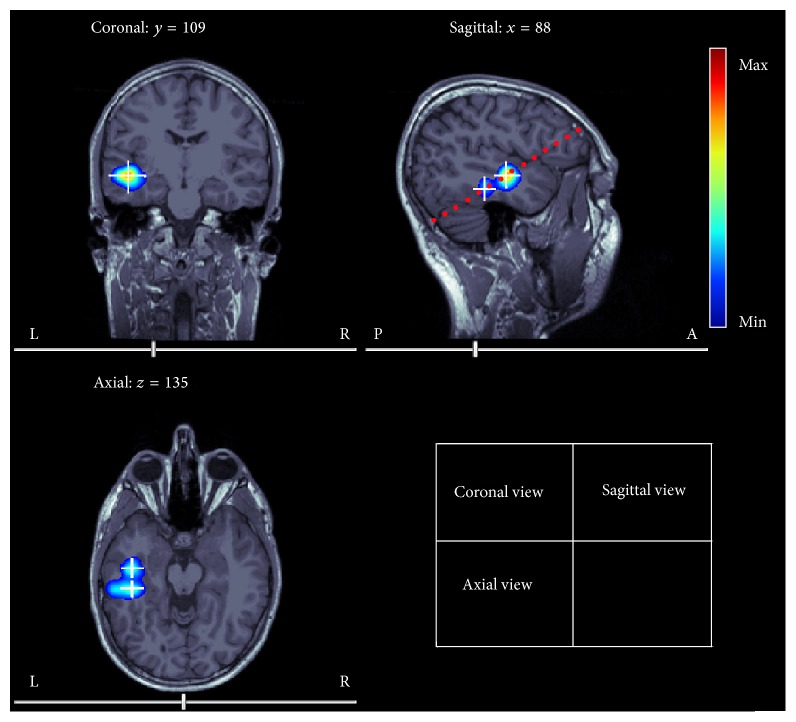
True source locations overlaid on T1 anatomical images. Two closely spaced simulated sources (indicated by white crosses) are located at (−5, 45, 40) mm and (5, 45, 40) mm, respectively. PW-INN-based source imaging result (obtained in the condition: real MEG noise; SNR = 18; *r* ^2^ = 0) was also shown as color-coded spatial patterns, where the color denotes the imaging index from (9). The dotted red line in the sagittal view indicates the plane of *z* = 40 mm.

We first tested how correlation and SNR modulate localization accuracy of source localization methods. Two equally strong sources were simulated: dipole 1 was located at (−5, 45, 40) mm with orientation (−0.6 −0.1, −0.7) and dipole 2 was at (5, 45, 40) mm with orientation (−0.9, 0.2, −0.3). These two sources were close to each other and intersource distance was 10 mm. The locations of these two dipoles were illustrated in Figure 2. The time courses of the two sources were both 10 Hz sine functions with a duration of 100 ms but with different phases. Note that we only simulate evoked neural activities at sources and do not include induced activities in our simulation. The sampling frequency of the simulated waveforms was 1200 Hz. The correlation coefficient (*r* ^2^) between the two sources was set to 1, 0.99, 0.97, 0.95, 0.9, 0.8, 0.7, 0.5, 0.3, or 0 by adjusting the phase difference between the time courses. Uncorrelated white Gaussian noise or real MEG noise was added to all data points scaled such that SNR was 10, 12, 14, 16, 18, 20, or 30. For each condition (a specific combination of SNR, noise type, and *r* ^2^), 100 trials of simulated evoked MEG responses were generated and the noise superimposed to each trial was either independently generated (for white noise) or randomly selected from available prestimulus MEG trials. All data analyses were performed using in-house MATLAB code.

#### 2.2.3. Reconstruction of Source Time Courses and FC Analysis

In this simulation, the simulated sources were configured almost the same way as in Section 2.2.2. We tested the performance of different source localization methods in reconstruction of source time courses and FC analysis under two stimulation cases. In Case 1, the phase difference between two sources is set to 18 degrees and intersource correlation coefficient is 0.95. In Case 2, the phase difference is set to 90 degrees and the intersource correlation coefficient is 0. After source localization, the time courses of identified sources were also reconstructed. Instead of plotting the reconstructed source time courses in three orientations (*xyz*-axes), in our results we only showed the norm of source time courses, which is called collective time courses (CTC).

Next, FC analysis was performed on reconstructed source time courses. Only sources with energy larger than 90% of the highest energy of the cost function were retained for subsequent FC analysis, and their time courses were reconstructed. A 6 Hz–14 Hz bandpass filter was applied on source time courses and then the phases of these filtered source time courses were extracted by Hilbert transformation for the subsequent FC analysis. Phase synchronization (PS) [20, 21] is used to measure the FC between reconstructed CTC at two source regions, because PS can effectively detect correlation between two signals even if they have phase difference [21]. PS is calculated as

(14) $$\begin{array}{c} PS\left(\;l,m\;\right)=\left|\;\langle \;e^{\Phi_{l}\left(t\right)-\Phi_{m}\left(t\right)}\;\rangle \;\right|, \end{array}$$

where Φ_*l*_(*t*) and Φ_*m*_(*t*) indicate the phases of the signals *l* and *m* at time *t*, respectively. A thresholding procedure was further applied on FC values to retain 10% strongest FC among all possible pairs of FC for a better visualization effect [21, 22].

## 3. Results

### 3.1. Source Imaging

**Figure 3:**
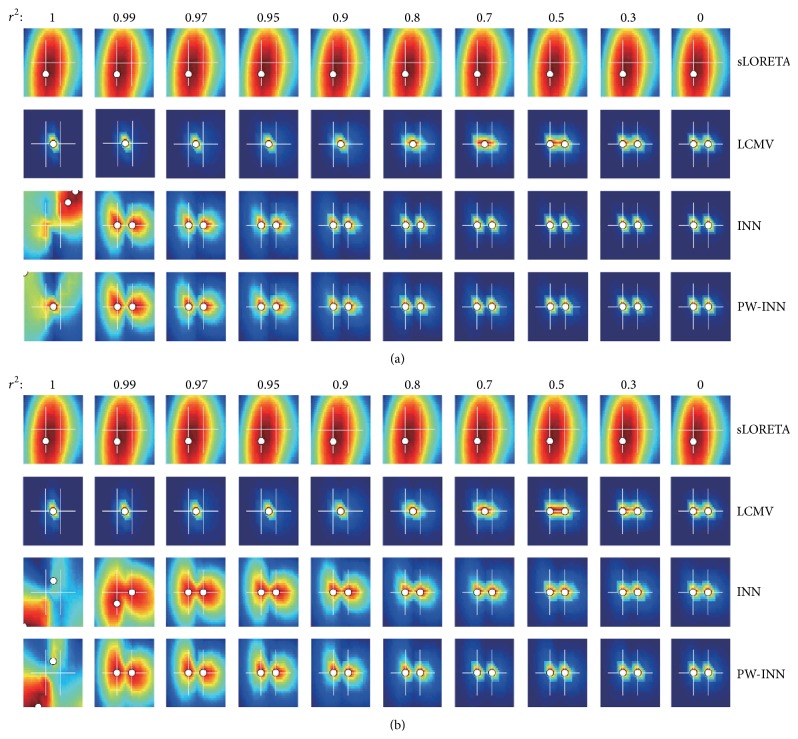
Performance comparison between sLORETA, LCMV beamformer, INN, and PW-INN in resolving closely spaced sources in presence of white noise (a) or real MEG noise (b). SNR is set to 18. Correlation coefficient (*r* ^2^) varied from 1 to 0. From leftmost column to rightmost, *r* ^2^ is sequentially set to 1, 0.99, 0.97, 0.95, 0.9, 0.8, 0.7, 0.5, 0.3, and 0. White crosses indicate true source locations and white dots indicate estimated source locations. All results are obtained by averaging 100 independent trials.

**Figure 4:**
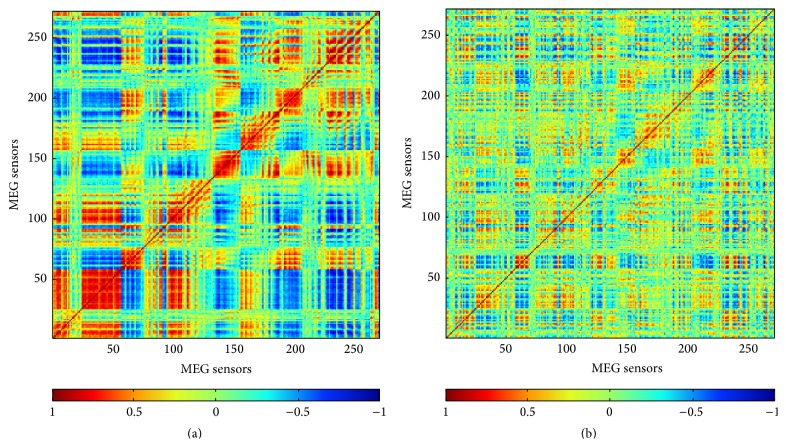
(a) Correlation coefficient matrix of real spontaneous MEG noise from a randomly selected trial. (b) Correlation coefficient matrix of real spontaneous MEG noise after being projected to a new space defined by the orthogonal complement of dominant eigenvectors of real MEG noise from another trial. The sum of absolute values of nondiagonal elements in (a) is 1.6 times larger than that in (b), implying that the spatial correlation between MEG noise can be decreased by the prewhitening operation. Colorbar indicates the values of correlation coefficient.

Figures 3(a) and 3(b) compare the performances of different source imaging methods under white noise and real MEG noise, respectively, when SNR = 18 dB. It can be clearly seen from Figure 3 that, regardless of which type of noise (white or real MEG) was added, (1) sLORETA cannot resolve these two closely spaced sources even if they are not correlated; (2) the performance of beamformer in resolving two closely spaced sources is decreased rapidly with the increase of correlation, and when *r* ^2^ ≥ 0.7 beamformer merged two sources into one equivalent source; (3) INN and PW-INN can still well resolve two sources even if correlation coefficient is as high as 0.97; (4) when *r* ^2^ = 1, which rarely happens in reality, all methods failed to resolve sources. By further comparing Figure 3(a) (under white noise) and Figure 3(b) (under real MEG noise), we can see that (1) real MEG noise increased the difficulty in resolving closely spaced sources for all methods; (2) PW-INN outperformed INN when two sources are highly correlated (*r* ^2^ = 0.99) only under real MEG noise. The source imaging results of PW-INN are also overlaid on anatomical images in Figure 2. The better performance of PW-INN over INN can be explained by Figure 4, which shows the spatial correlation between real MEG noise of different sensors before and after prewhitening. It can be clearly seen that MEG noise exhibited very strong spatial correlation, but the spatial correlation can be greatly lowered by the prewhitening operation. For example, the sum of absolute values of nondiagonal elements in the correlation matrix, which represents the overall degree of cross-sensor correlation, before prewhitening is 1.6 times larger than that after prewhitening.

We also compared these source imaging methods under various levels of noise (SNR = [10,12,14,16,18,20, or 30]). In general, as SNR increased, all methods can resolve these two closely spaced sources with higher accuracy. The performance difference between different methods under other SNR values is similar to the observation under a SNR of 18 dB. Due to page limitation, we only showed the results under SNR = 18 dB in Figure 3. It should also be noted that the prewhitening operation adopted by PW-INN is also used for sLORETA and beamformer, but we did not display the results here because prewhitening did not significantly improve the performance of sLORETA or beamformer.

### 3.2. Reconstruction of Source Time Courses

**Figure 5:**
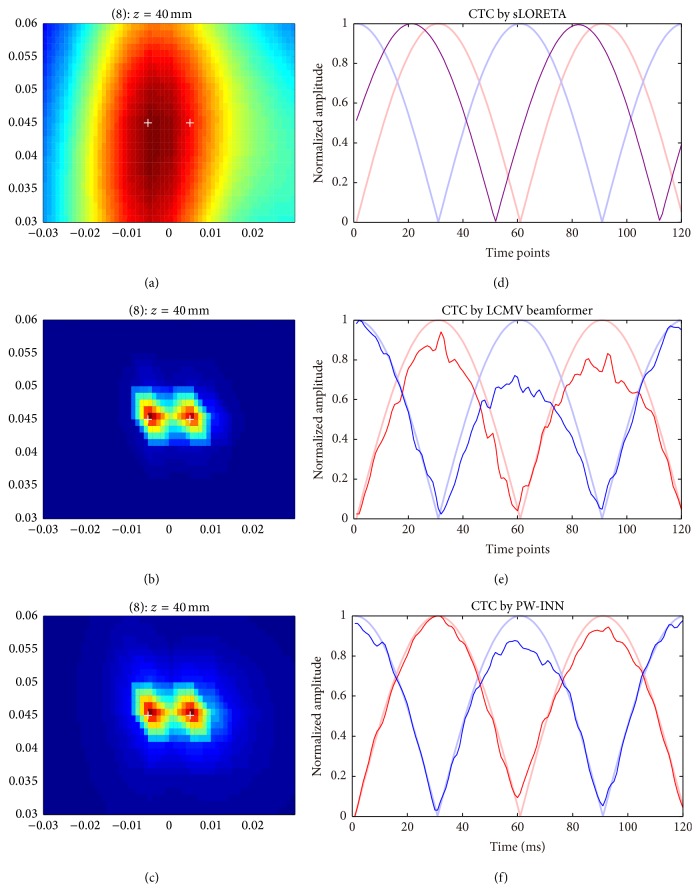
Source reconstruction using sLORETA, LCMV beamformer, and PW-INN. Source locations were the same as those in Figure 2. Real MEG noise was added such that SNR = 18. Two simulated source time courses have phase difference of 90 degrees. The correlation *r* ^2^ between sources is 0. (a)–(c) Source distribution estimated from sLORETA (a), LCMV beamformer (b), and PW-INN (c). White crosses indicate the true source locations. (d)–(f) Source time courses at the peak locations estimated from sLORETA (d), LCMV beamformer (e), and PW-INN (f). The time courses in (d)–(f) are the norm of the original time courses over *xyz*-axis (i.e., collecting time courses, CTC). Bold lines in light red and light blue indicate the true source time courses and thin lines in red and blue indicate the estimated ones. The purple color line in (d) indicates the time course from the wrongly estimated equivalent source.

**Figure 6:**
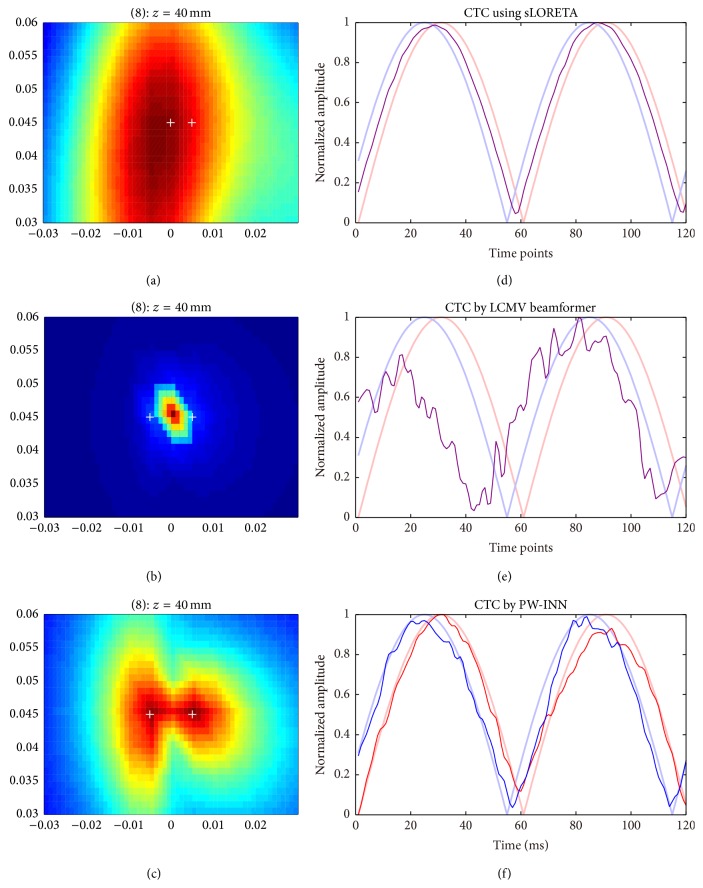
Source reconstruction using sLORETA, LCMV beamformer, and PW-INN. Source locations were the same as those in Figure 2. Real MEG noise was added such that SNR = 18. Two simulated source time courses have phase difference of 18 degrees. The correlation *r* ^2^ between sources is 0.95. (a)–(c) Source distribution estimated from sLORETA (a), LCMV beamformer (b), and PW-INN (c). White crosses indicate the true source locations. (d)–(f) Source time courses at the peak locations estimated from sLORETA (d), LCMV beamformer (e), and PW-INN (f). The time courses in (d)–(f) are the norm of the original time courses over *xyz*-axis (i.e., collecting time courses, CTC). Bold lines in light red and light blue indicate the true source time courses and thin lines in red and blue indicate the estimated ones.

Figure 5 showed the source imaging results and the corresponding source time courses estimated using sLORETA, beamformer, and PW-INN, respectively, when there was no correlation between two simulated sources (*r* ^2^ = 0) and real MEG noise of 18 dB is added. For comparison, all time courses in Figure 5 were normalized by the corresponding maximum values. The results of INN are not included here for comparison, because the previous section has shown that the new PW-INN method has better or at least comparable performance than INN. It can be clearly seen from Figure 5 that beamformer and PW-INN accurately identified two sources and recovered the source time courses, while sLORETA failed to do so. As a comparison, Figure 6 showed source imaging and reconstruction results with a correlation of *r* ^2^ = 0.95. We can see that when the correlation between two sources is high both sLORETA and beamformer wrongly placed an equivalent source between two true sources, and, thus, they can only estimate one equivalent source time course. Under this testing condition, the proposed PW-INN method was still able to accurately identify two sources and to satisfactorily recover source time courses.

#### 3.2.1. Source-Level Functional Connectivity Analysis

**Figure 7:**
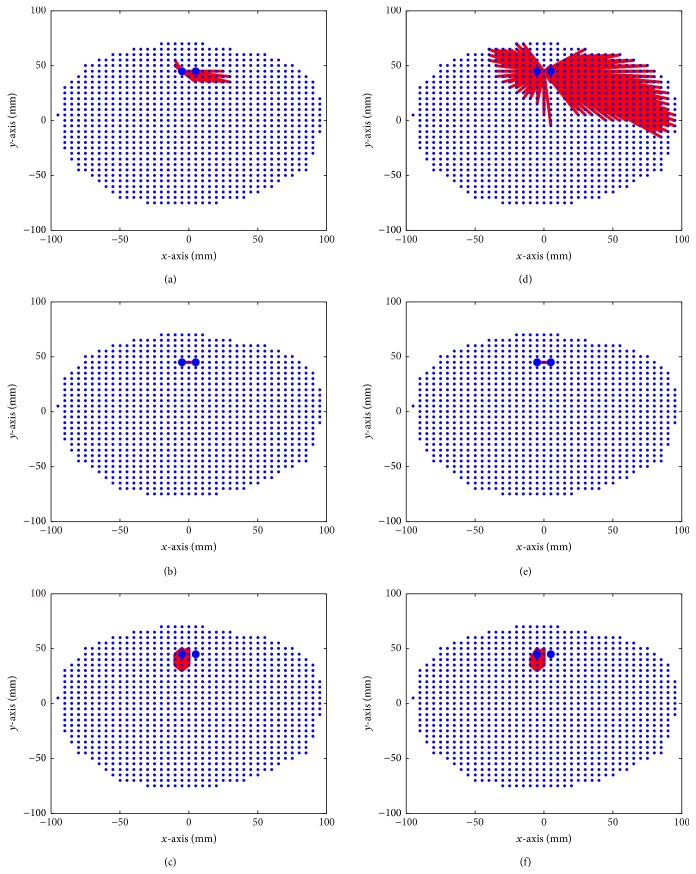
FC graphs based on source reconstruction results using sLORETA, LCMV beamformer, and PW-INN. Real MEG noise was added such that SNR = 18. Small blue dots indicate brain volume grid points, and large blue dots indicate true source locations. (a)–(c) FC graphs estimated from sLORETA (a), LCMV beamformer (b), and PW-INN (c), when *r* ^2^ = 0 between two sources. (d)–(f) FC graphs estimated from sLORETA (d), LCMV beamformer (e), and IINN (f), when *r* ^2^ = 0.95 between two sources.

Once source locations and time courses were readily estimated, FC analysis can be easily implemented.  Figure 7 showed the FC graph based on reconstruction results of three methods, sLORETA, beamformer, and PW-INN, when two closely spaced sources have two different correlation coefficients, *r* ^2^ = 0 and 0.95. It can be obviously seen that only PW-INN can accurately identify true FC patterns, while FC graphs based on sLORETA and beamformer have many spurious connections. We can also see that FC graphs of sLORETA are relatively consistent (though incorrect) for sources with different correlation coefficients, but FC graphs of beamformer show more spurious connections when the correlation between two true sources is large.

## 4. Discussion and Conclusion

### 4.1. Closely Spaced Source Analysis under Real MEG Noise

This study intensively investigated the performance of MEG source localization methods for closely spaced source in the presence of real MEG noise and white noise.

Simulated signals with realistic characteristics are important for developing and evaluating new methods. In our previous work [15], the good performance of our proposed INN method in localizing closely spaced sources was validated in the presence of white noise only. However, the INN method is based on the assumption of uncorrelated white noise, but real MEG noise has largely different characteristics (e.g., the degree of correlations between sensors) with white noise. Figure 4 clearly showed that there are large correlations between spontaneous MEG noise at different sensors. In order to evaluate source analysis methods with more realistic signals, we used spontaneous MEG recordings to simulate real MEG noise. Although INN still outperformed beamformer and sLORETA, its performance was degraded under real MEG noise, as compared with under white noise. The newly developed PW-INN can decorrelate MEG signals, so that it can achieve better performance than INN in presence of real correlated MEG noise.

### 4.2. MEG-Based Source-Level Connectivity Analysis

Identifying connectivity between cortical sources using MEG has gained increasing popularity. Correct source localization is the prerequisite of accurate reconstruction of source time courses, which is again the prerequisite for precise inference of source-level connectivity. Many studies based on the classical beamformer have made much progress in identifying physiological and pathological MEG-based source-level FC ([23] and a review in [8]). However, as discussed earlier, the beamformer has difficulty in separating closely spaced sources, especially in the case of strong intersource connectivity. As shown in Figure 5, the beamformer estimated one spurious source between two true sources, when these two true sources are highly correlated. Apparently, in such a case, source-level connectivity analysis will fail. The proposed PW-INN method inherits the advantage of the INN method, so that it can perform well in localizing highly correlated closely spaced sources and reconstructing their time courses. Therefore, based on the source reconstruction results of PW-INN, connectivity between sources can be correctly identified.

### 4.3. Significance and Implications

The present study is relevant to many important research topics in basic and clinical neuroscience, because the proposed MEG source analysis method can achieve a high spatial-temporal characterization of cortical source activity and connectivity. Nowadays, fMRI is the most popular imaging tool to construct voxel-level brain networks, because of its high spatial resolution. However, fMRI can only provide indirect measures of neural activities with a low temporal resolution, which make it not an ideal tool to investigate the highly dynamic organization of the human brain. MEG can directly detect and track neural electromagnetic activity with high temporal precision, so the proposed PW-INN and the subsequent connectivity analysis can identify cortical networks with a higher temporal resolution than fMRI, which is particularly suitable for tracking dynamic connectivity changes in sensory and cognitive experiments.

Constructing MEG brain network is attracting more and more interests. Currently, there is a huge body of publications that use sLORETA and beamformer to extract source time courses for FC analysis (i.e., [24, 25]). But, as shown in this study, sLORETA and beamformer cannot effectively resolve closely spaced sources, so that they can only be used for constructing a brain network with coarse spatial resolution (e.g., region-level FC or lobe-level FC). Another type of approach to construct MEG networks is the atlas-based MEG analysis, which can estimate FC between different brain regions or lobes [26, 27]. Thus, the spatial resolution of this atlas-based MEG networks is still coarse. So far, important information conveyed by MEG and underlying interconnection between closely spaced small cortical areas (at voxel level) [28] are generally overlooked. Actually, these voxel-level small brain regions have slightly different activities with their neighboring regions, which carry useful information for understanding cortical processing and organization. Identifying the connectivity between primary auditory cortex and secondary auditory cortex using MEG could greatly increase our understanding of auditory information processing in the brain, but it is also very challenging. Unlike previous region- or lobe-based MEG connectivity analysis, the proposed PW-INN method can resolve closely spaced sources, which enable constructing a voxel-level brain network.

In summary, this study is an important step towards a high spatial-temporal characterization of cortical activity and connectivity. In future, we plan to build a high-spatial-resolution voxel-level whole-brain connectome using MEG and apply this MEG connectome in practical and clinical applications (e.g., to identify changes of MEG connectome for studying neural plasticity).
