# Corrigendum to “Closely Spaced MEG Source Localization and Functional Connectivity Analysis Using a New Prewhitening Invariance of Noise Space Algorithm”

**DOI:** 10.1155/2016/7473217

**Published:** 2016-09-14

**Authors:** Junpeng Zhang, Yuan Cui, Lihua Deng, Ling He, Junran Zhang, Jing Zhang, Qun Zhou, Qi Liu, Zhiguo Zhang

**Affiliations:** ^1^Department of Medical Information Engineering, School of Electrical Engineering and Information, Sichuan University, Chengdu 610065, China; ^2^School of Chemical and Biomedical Engineering, School of Electrical and Electronic Engineering, Nanyang Technological University, Singapore 639798; ^3^School of Humanities and Information Management, Chengdu Medical College, Chengdu 610083, China

 In the article titled “Closely Spaced MEG Source Localization and Functional Connectivity Analysis Using a New Prewhitening Invariance of Noise Space Algorithm” [1], there was an error in the legend of Figure 7, which should be corrected as follows.

## Figures and Tables

**Figure 7:**
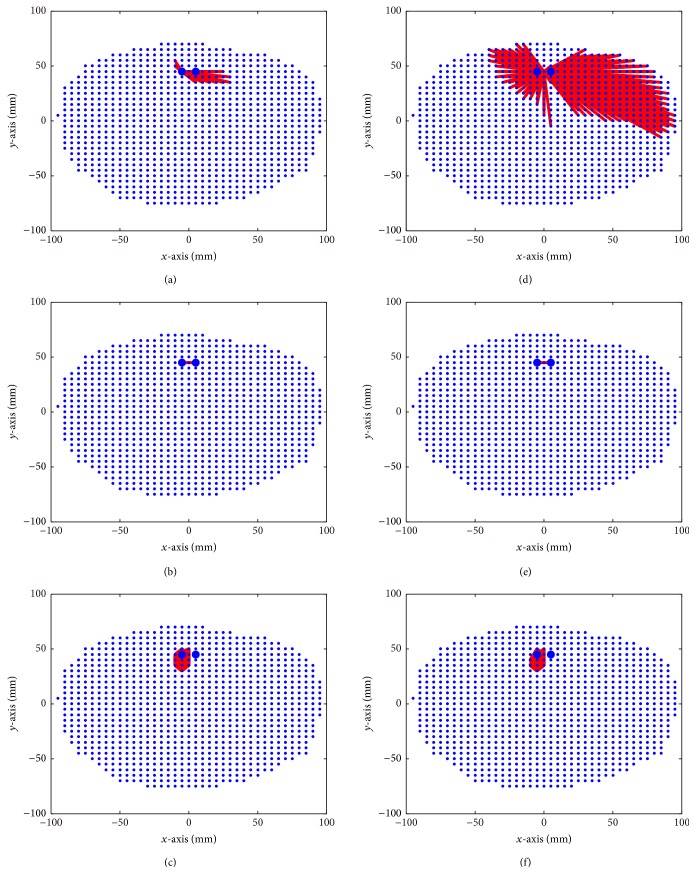
FC graphs based on source reconstruction results using LCMV beamformer, PW-INN, and sLORETA. Real MEG noise was added such that SNR = 18. Small blue dots indicate brain volume grid points, and large blue dots indicate true source locations. (a)–(c) FC graphs estimated from LCMV beamformer (a), PW-INN (b), and sLORETA (c) when *r* ^2^ = 0 between two sources. (d)–(f) FC graphs estimated from LCMV beamformer (d), PW-INN (e), and sLORETA (f), when *r* ^2^ = 0.95 between two sources.
